# Benign Pneumoperitoneum Following Mitral Valve Replacement

**DOI:** 10.7759/cureus.53216

**Published:** 2024-01-30

**Authors:** Ali Tariq Alvi, Luis E Santiago, Murali Shankar, Pallavi Aneja

**Affiliations:** 1 Internal Medicine, HCA Florida Westside Hospital, Plantation, USA; 2 Internal Medicine, HCA Florida Northwest Hospital, Margate, USA

**Keywords:** free air in abdomen, heart surgery, post-op complications, mitral valve replacement, benign pneumoperitoneum

## Abstract

The pneumoperitoneum refers to the presence of free air inside the abdominal cavity. This finding is usually a sequela of a gastrointestinal tract perforation. Still, in rare instances, it can present after cardiac surgery due to the proximity of the peritoneal cavity and pericardium, allowing air to enter the peritoneal cavity. Our patient was a 63-year-old female who initially presented for revision of the mitral valve replacement. A chest X-ray on postoperative day 13 revealed a 6.6 cm lucency under the right diaphragm suggestive of pneumoperitoneum. She was discharged after serial chest X-rays revealed a decrease in the size of the pneumoperitoneum. Twelve days later, our patient was readmitted, as another chest X-ray revealed that the size of the pneumoperitoneum was again increasing. An endoscopy was performed, but it did not reveal any lesions or etiology that would lead to a leak from the gastrointestinal tract. Finally, due to the benign nature of the pneumoperitoneum and the decrease in its size over the following days, we opted for conservative management, and she was discharged again. This case emphasizes the rare occurrence of benign pneumoperitoneum post-mitral valve surgery. While surgery may not always be required for asymptomatic cases, careful vigilance post-cardiac surgery remains crucial to detect potential abdominal complications promptly.

## Introduction

Benign pneumoperitoneum refers to the pneumoperitoneum without accompanying peritonitis or the presence of asymptomatic intrabdominal free air [1]. Pneumoperitoneum occurs from perforation of abdominal organs in 85-95% of cases, necessitating urgent surgical intervention. However, in the remaining 5 to 15 instances, pneumoperitoneum does not stem from perforation but instead originates from another non-emergent source [2]. The development of pneumoperitoneum following surgery of the mitral valve or other cardiac surgeries is rare, with only a few cases previously reported. During cardiac surgery involving sternotomy, the proximity of the peritoneal cavity, pericardium, and diaphragm can lead to an abrupt diaphragmatic opening, allowing air to enter the peritoneal cavity [3]. Here, we present a case of persistent benign pneumoperitoneum developing after revision surgery of the mitral valve.

## Case presentation

**Figure 1:**
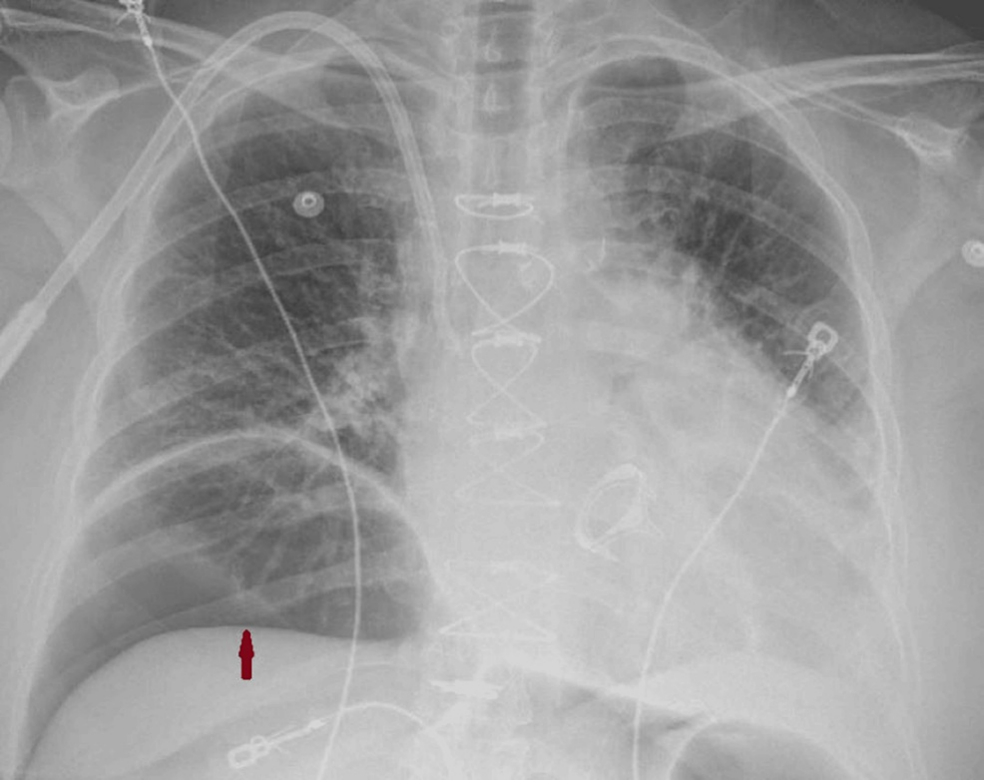
Supine chest X-ray demonstrating an area of lucency underneath the right hemidiaphragm representing intra-abdominal free air (red arrow)

We describe a 63-year-old female with a past medical history of hyperlipidemia, hypothyroidism, mitral regurgitation, and bioprosthetic mitral valve replacement with conventional sternotomy two years ago who presented for revision surgery of the mitral valve. The postoperative hospital course was complicated, with renal failure requiring hemodialysis. On day 13, a chest X-ray revealed increased lucency underneath the right hemidiaphragm concerning pneumoperitoneum measuring up to 6.6 cm (Figure 1).

**Figure 2:**
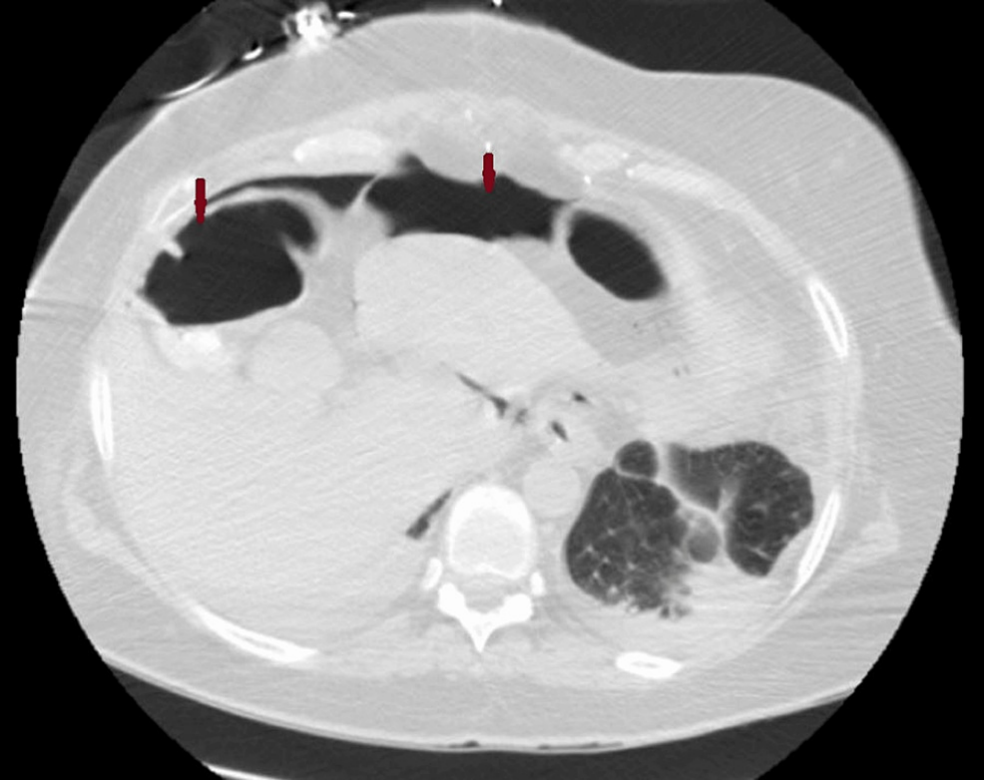
Axial view of computed tomography (CT) chest demonstrating free air in the peritoneal cavity (red arrows)

A computed tomography (CT) scan of the chest showed small bilateral pleural effusions and large volume pneumoperitoneum (Figure 2).

**Figure 3:**
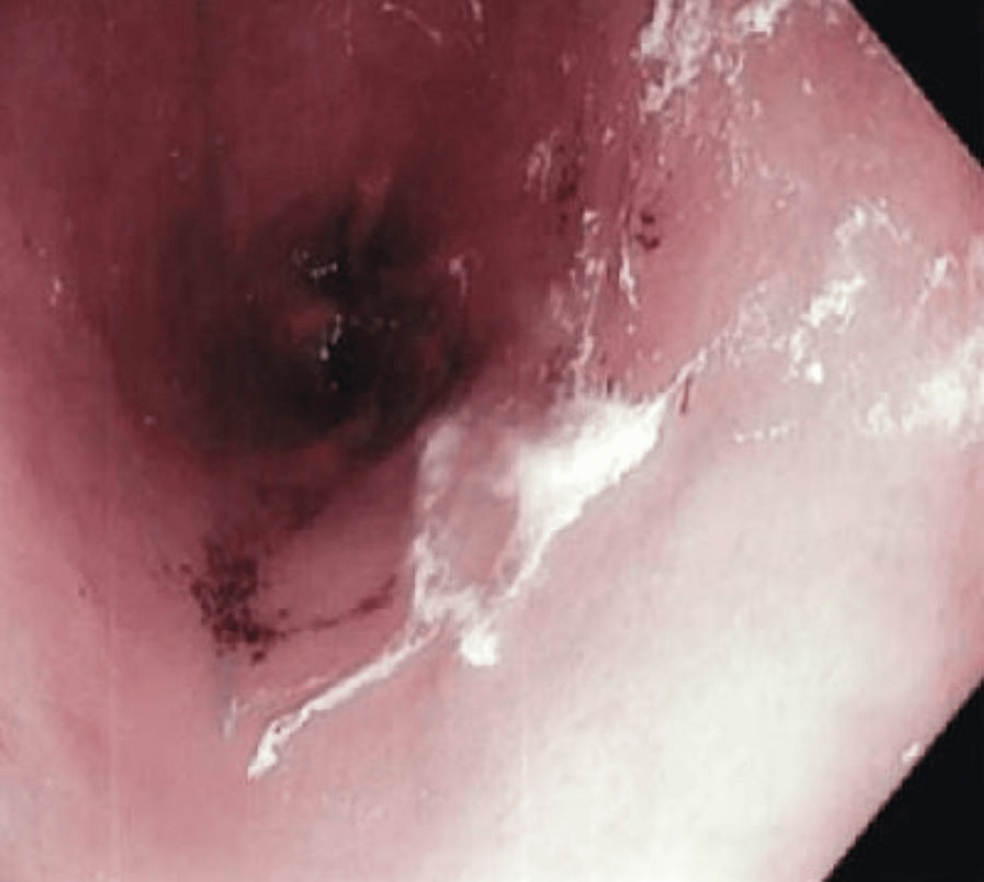
Upper endoscopy showing antral gastritis with some epithelial hemorrhages

The patient denied any symptoms at that time, and on examination, her abdomen was soft, non-distended, and nontender. She was observed clinically with serial chest X-rays, which showed improvement of pneumoperitoneum on the following days. Therefore, the patient was finally discharged home on postoperative day 17. A repeat chest X-ray was done 12 days later, which showed a worsening of the pneumoperitoneum. Thus, she was readmitted to our facility for further evaluation. She again denied any symptoms, and vital signs were also stable. Her laboratory investigations revealed a white blood cell (WBC) count of 7900/mm^3^, hemoglobin of 8.8 g/dl, platelet count of 296000/ul, sodium of 135 mmol/L, potassium of 3.5 mmol/L, chloride of 95 mmol/L, bicarbonate of 35 mmol/L, blood urea nitrogen of 14 mg/dl, creatinine of 4.4 mg/dl, calcium of 9 mg/dl, total bilirubin of 0.4 mg/dl, aspartate aminotransferase (AST) of 28 units/L, and alanine aminotransferase (ALT) of 12 units/L. The abdomen was soft, non-tender, and non-distended, with no rebound or guarding on physical examination. An upper gastrointestinal series (UGI) was performed, which revealed no evidence of obstruction or leak in the setting of persistent pneumoperitoneum. Endoscopy was undertaken to evaluate any abnormalities in the upper gastrointestinal tract that could explain the persistent leak. It did not show any ulcers or lesions explaining the etiology of pneumoperitoneum, only revealing antral gastritis with some subepithelial hemorrhages (Figure 3).

Due to the benign nature of the pneumoperitoneum and the decrease in its size over the following days, we opted for conservative management.

## Discussion

Most non-surgical or benign pneumoperitoneum cases arise either as a complication of medical intervention or as a complication related to some procedure. Among these, the common etiologies include retention of postoperative air with a prevalence ranging from 25% to 60%, endoscopic procedures of the gastrointestinal tract with a prevalence ranging from 0.3% to 25%, placement of peritoneal dialysis catheter with a prevalence of 10% to 34%, mechanical ventilation, pneumothorax, and cardiopulmonary resuscitation [2]. Benign pneumoperitoneum is characterized by the presence of asymptomatic intra-abdominal free air and manifests on chest X-ray as a distinctive radiolucency beneath the diaphragm or on abdominal X-ray as a radiolucency in the superior location [1,2].

Surgical treatment may not be necessary in all cases of pneumoperitoneum, as it is not invariably associated with bowel injury [2-4]. Simultaneously, it is crucial to prevent any delay in conducting a laparotomy when there is a suspected bowel injury. Therefore, to avoid unnecessary laparotomy, it is essential to consider recent events that could lead to spontaneous pneumoperitoneum [3].

Complications within the abdomen can arise in 0.5% to 3% of individuals undergoing open heart surgeries, with mortality rates ranging from 14.5% to 100% [3,5-7]. The common gastrointestinal complications following cardiac surgeries include intestinal infarction, gastrointestinal hemorrhage, and acute pancreatitis [3,8]. The development of pneumoperitoneum is unusual, with only a few cases previously reported [3,9,10]. The etiologies of pneumoperitoneum can be diverse; however, in the context of cardiac surgery, it can result from diaphragmatic damage during sternotomy or during chest tube placement [3].

## Conclusions

The case presented here highlights the rare occurrence of benign pneumoperitoneum following the revision of a mitral valve replacement. While pneumoperitoneum is commonly associated with perforation of the gastrointestinal tract necessitating urgent surgical intervention, it's crucial to recognize instances where it arises from other sources, as in this case. The diagnosis can be challenging, often necessitating thorough clinical evaluation and imaging studies. This case underscores the importance of a comprehensive approach to diagnosis involving serial imaging, endoscopic evaluation, and clinical observation to determine the benign nature of pneumoperitoneum and guide appropriate management decisions.

While surgical intervention may not be necessary in all cases of pneumoperitoneum, especially when it is benign and asymptomatic, vigilance is essential to promptly identify and address any potential complications arising within the abdomen, particularly following cardiac surgeries. Understanding the diverse etiologies of pneumoperitoneum, including its rare occurrences after cardiac procedures, is crucial to implementing the appropriate management strategies, ultimately ensuring optimal patient care and outcomes.
